# Exploring women’s experiences of participation in shared decision-making during childbirth: a qualitative study at a reference hospital in Spain

**DOI:** 10.1186/s12884-021-04070-3

**Published:** 2021-09-17

**Authors:** María López-Toribio, Paulina Bravo, Anna Llupià

**Affiliations:** 1 Preventive Medicine and Epidemiology Department, Hospital Clínic, Barcelona, Spain; 2 Faculty of Medicine and Health Sciences, University of Barcelona, Barcelona, Spain; 3 School of Nursing, Pontificia Universidad Católica de Chile, Santiago, Chile; 4 Centro Núcleo Milenio Autoridad y Asimetrías de Poder / Millennium Nucleus Center Authority and Power Asymmetries, Santiago, Chile; 5 Barcelona Institute for Global Health (ISGlobal), Barcelona, Spain

**Keywords:** Shared decision-making, Patient participation, Birth plan, During childbirth, Delivery rooms, Parturition

## Abstract

**Background:**

Women’s engagement in healthcare decision-making during childbirth has been increasingly emphasised as a priority in maternity care, since it increases satisfaction with the childbirth experience and provides health benefits for women and newborns. The birth plan was developed as a tool to facilitate communication between health professionals and women in Spain, but their value in routine practice has been questioned. Besides, little is known about women’s experiences of participation in decision-making in the Spanish context. Thus, this study aimed to explore women’s experiences of participation in shared decision-making during hospital childbirth.

**Methods:**

An exploratory qualitative study using focus groups was carried out in one maternity unit of a large reference hospital in Barcelona, Spain. Participants were first-time mothers aged 18 years or older who had had a live birth at the same hospital in the previous 12 months. Data collected were transcribed verbatim and analysed using a six-phase inductive thematic analysis process.

**Results:**

Twenty-three women participated in three focus groups. Three major themes emerged from the data: “Women’s low participation in shared decision-making”, “Lack of information provision for shared decision-making”, and “Suggestions to improve women’s participation in shared decision-making”. The women who were willing to take an active role in decision-making encountered barriers to achieving this and some women did not feel prepared to do so. The birth plan was experienced as a deficient method to promote women’s participation, as health professionals did not use them. Participants described the information given as insufficient and not offered at a timely or useful point where it could aid their decision-making. Potential improvements identified that could promote women’s participation were having a mutually respectful relationship with their providers, the support of partners and other members of the family and receiving continuity of a coordinated and personalised perinatal care.

**Conclusion:**

Enhancing women’s involvement in shared decision-making requires the acquisition of skills by health professionals and women. The development and implementation of interventions that encompass a training programme for health professionals and women, accompanied by an effective tool to promote women’s participation in shared decision-making during childbirth, is highly recommended.

## Background

Shared decision-making (SDM) has been identified as the ideal approach to promote patients’ involvement in health decisions [1, 2]. SDM is a process in which clinicians and patients consider available information about the medical problem and work together to make a decision taking into account the patient’s preferences and values [3]. SDM has proven to increase satisfaction and reduce decisional conflict, as well as to improve health outcomes [4, 5]. Particularly in maternity care, SDM has been associated with higher satisfaction of childbirth experience among women and increased involvement in decision-making [6–8]. Additionally, the health benefits of women’s participation in decision-making during childbirth have been associated with reductions in perinatal depressive symptoms, preterm birth, and low birthweight rates [9]. In the context of a growing number of facility-based childbirths, where over-medicalisation and inappropriate use of interventions may occur [10], women’s engagement in SDM has been increasingly emphasised as a priority in maternity care [11].

Birth plans were developed as a tool to facilitate communication and promote women’s participation in decision-making during childbirth [12]. However, their value has been widely questioned, since birth plans can irritate professionals and create unmet expectations in women if the plan is not accomplished. Indeed, a birth plan could serve to hinder communication rather than promote it among care providers and women [13–16]. Moreover, it has been reported that women who used a birth plan were less satisfied and felt less in control during childbirth in comparison with women without a birth plan [17]. The Spanish Ministry of Health officially introduced the birth plan as a recommendation in 2008 [18] and a birth plan sample template was published in 2012 [19]. It has the format of a checklist where women can select their preferences regarding support, physical space, and medical interventions during childbirth and immediate postpartum. Additionally, each hospital can offer their own birth plan template with its own adapted options. Frequently, birth plans are offered to women during antenatal care and women should hand it in at the Hospital when they are admitted for childbirth, as the birth plan is a paper-based document, and it is not currently digitalised in the National Health System. A review of the birth plans offered by Catalonian public hospitals stated that the plans were out-of-date and that some could perpetuate unrecommended practices, such as optional enemas or perineal shaving, rather than giving voice to women’s preferences and needs [20]. As an alternative, some authors have advocated for a ‘birth partnership’ between care providers and women that goes beyond the checklist of limited choices presented on birth plans [13, 21]. According to this shift towards a ‘birth partnership’, the creation of a trustworthy and supporting relationship through effective communication between women and care providers is the cornerstone that ensures respect for women’s values and preferences and promotes their participation in SDM during childbirth. This approach is also desirable in high-risk pregnancies, where safety concerns could be associated with more uncertainty and anxiety among pregnant women [22].

In Catalonia, at the time of the study, women with low- and medium-risk pregnancies receive antenatal care by midwives at community health centres. In the third trimester of pregnancy, all women have an appointment with a hospital midwife at the hospital where they will give birth. On the other hand, women with high- or very high-risk pregnancies receive antenatal care at hospitals, mainly by obstetricians. During antenatal care, women are more frequently attended by the same midwife or obstetrician, which facilitates continuity of care. However, constant shifts of personnel at the hospital delivery room hinder continuity of care during the attendance of childbirth and immediate postpartum.

Although some studies have evaluated women’s experiences of choice during childbirth [23], most of them have focused on the election of the place of birth [24]. In addition, to the best of our knowledge, there have been no studies in Spain which have comprehensively explored women’s experiences of participation in SDM throughout the continuum that comprises prepartum, birth, and immediate postpartum [25]. This study aimed to explore women’s experiences of participation in shared decision-making during hospital childbirth.

## Methods

An exploratory qualitative study using focus groups was carried out in one maternity unit of a large reference hospital in Barcelona, Spain. Ethical approval was granted by the Clinical Research Ethics Committee of the Hospital Clinic of Barcelona (Reference number: HCB/2017/1069). This study complied with the basic ethical principles contained in the 2013 Helsinki Declaration [26].

### Aim and research questions

This study aimed to explore women’s experiences of participation in SDM during hospital childbirth. Specifically, two research questions were stated: 1) What were the barriers and facilitators to women’s involvement in SDM during hospital childbirth? 2) What were the opportunities for improvement regarding participation in SDM? This study was conducted and reported according to the guidelines of the Consolidated Criteria for Reporting Qualitative Research (COREQ) [27].

### Study site and population

In Catalonia, the average number of children per woman was 1.27 and the average maternal age reached 31.2 years for the year 2019 [28]. In the same year, 99.4% of childbirths were attended at care facilities, principally hospitals, as home birth is not offered by the Spanish National Health System [29]. The Hospital Clínic of Barcelona (HCB) provides the highest level of complexity of care at the obstetric unit and attends more than 3000 births per year, almost 70% of which are considered high or very high-risk. The risk classification used at the HCB is based on the recommendations made by the Department of Health of Catalonia in its pregnancy care guideline, published in 2018 [30]. This guideline defines four levels of risk during pregnancy: low-risk, medium-risk, high-risk and very high-risk. At HCB, childbirths are generally attended by midwives except when complications occur or women have been diagnosed with high- or very high-risk pregnancies, in those cases obstetricians attend the childbirth with the support of midwives.

### Participant recruitment

Inclusion criteria were first-time mothers aged 18 years or older who had given birth at HCB in the previous 12 months. Only first-time mothers were included because previous experiences of childbirth could improve their involvement in SDM and their sense of control [31]. Exclusion criteria were the mother having given the newborn up for adoption, having had a pregnancy which resulted in a stillbirth, or feeling uncomfortable/not emotionally prepared to share their birth experience in focus groups. The sample was built using convenience sampling, and some women were enrolled through snowball sampling.

Women were recruited in-person at the obstetric unit of HCB and at postpartum groups of two community health centres in the hospital area. Information regarding the research question and the aim of the study was provided face-to-face by one researcher (ML). Participants who expressed an interest in the research received a participant information sheet and a consent form to take home and read before deciding if they wished to participate. Later, all participants were contacted by telephone and invited to take part in scheduled focus groups. Participation was voluntary and no financial incentive was offered. All women signed and returned an informed consent form prior to their participation in a focus group and had the opportunity to ask the researchers any questions they had.

### Data collection

Focus groups were considered the most appropriate qualitative technique due to the exploratory nature of the research question, as well as the richness of discourse elicited on account of the synergistic effects that result from interactions among participants [32].

From September to December 2018, three focus groups were conducted by two female researchers (ML, as moderator and AL, as observer). To facilitate participant attendance, these varied in terms of time and location; two groups were conducted at the HCB, and another at a community health centre. Women were invited to attend focus groups with their babies if they wished to. The languages used were Spanish and Catalan. Participants’ sociodemographic characteristics and type of childbirth were obtained from hospital records, as approved by the Clinical Research Ethics Committee. There was no relationship between the women and the researchers prior to the focus groups. To minimise the possible influence of researchers’ preconceptions on the development of the focus groups and the analysis, a semi-structured topic guide was used and the three researchers made an exercise of bracketing as recommended by Tufford and Newman along the research process [33]. The topic guide (Table 1) was designed based on the literature review findings, previous experience of researchers in the field and suitability to the research question. The methodology described by Krueger and Cassey was followed to elaborate it [34].

**Table 1:** Semi-structured topic guide used in focus groups

Topic	Questions
**Experience of birth care**	1. What did you think was useful and not useful during the antenatal classes for yourself and for your partner?
	2. What skills do you think a woman and her partner should learn for childbirth and immediate postpartum?
	3. How did you feel during childbirth? How do you think your partner felt? What role do you think healthcare professionals had in making you feel this way?
	4. What do you think about birth plans? Did you use one?
**Quality of information and treatment received**	5. What is your opinion about the information that healthcare professionals gave you during childbirth?
	6. How would you describe the treatment that you received from healthcare professionals in childbirth?
**Opportunities for improvement regarding participation**	7. What do you consider that healthcare professionals should know about you to provide you the best quality of care during childbirth?
	8. What skills do you believe that a healthcare professional should have to provide you the best quality of care during childbirth?
	9. If you think of your childbirth and the decisions you made, how do you think this participation could be improved?
	10. What do you think that healthcare professionals should do to promote women’s participation in decision-making during childbirth?

Focus groups were audio-recorded and transcribed verbatim by one researcher (ML). Transcripts were anonymised to guarantee confidentiality and texts were verified three times against the audio, by two researchers independently, to assure accuracy of transcription. During focus groups, the observer made notes that were included in the analysis. After the third focus group, the research team considered that data saturation had been reached, as no new information was identified [35].

### Data analysis

The data were analysed following the six-phase inductive thematic analysis process described by Braun and Clarke [36], as follows: 1) becoming familiar with the data by reading and re-reading the entire dataset and taking notes of initial ideas; 2) generating initial codes and collating the data relevant to each code, carried out by two researchers independently; 3) compiling the codes into potential themes, carried out by the same two researchers independently, and then discussing them until consensus was reached; 4) reviewing the themes in relation to the coded extracts and the entire dataset to ensure they were consistent with the data; 5) refining and naming themes using words and phrases; 6) selection of data extracts to illustrate the themes and relating the analysis back to the research question and the literature. A third researcher validated and supervised each of the steps and the final structure of themes. Throughout the thematic analysis, a process of triangulation was performed by the three authors to ensure consistency in analysis and findings. The first two phases of the analysis were supported by Atlas.ti v7 software.

After analysis, in-person member checking was designed based on the Synthesized Member Checking methodology [37]. All participants were invited to a face-to-face session at the HCB where two of the researchers (ML and AL) presented the findings of the study. Then, ML moderated a discussion with the following questions: 1) Are you surprised by anything that we have presented? 2) Does it correspond to your experience? 3) Is there something that you miss or that you would add? 4) Have we presented something that you do not agree with? Six women attended and their comments were collected and added to the final analysis.

## Results

From September to December 2018, a total of 23 first-time mothers participated in three focus groups (with 6, 7, and 10 participants, respectively); each one lasted between 90 and 120 min. Sociodemographic characteristics of participants and the type of childbirth they experienced are described in Table 2. The majority of women were in the age range of 30 to 40 years old, had been born in Spain and had completed university studies. Older and higher educated women were over-represented in comparison with the average for Catalonia. Almost 60% of the sample (*n* = 13) had a high-risk or a very high-risk pregnancy, 11 of them had an induction of labour. Given that nearly 70% of the births attended at the HCB are of women diagnosed with high- or very high-risk pregnancies, this sample could be considered representative of the population attended at the HCB in this respect, but not representative of the whole population. In total, 60% of participants (*n* = 14) had onset of labour by induction and all but one woman used epidural anaesthesia.

**Table 2:** Sociodemographic characteristics and type of childbirth of focus group participants (*n* = 23)

	Women (***n*** = 23)	
	n	%
**Age in years (**$\overline{\boldsymbol{x}}=$**35.45 (***σ*= **6.23))**		
20–29	2	9
30–40	17	74
41–46	4	17
**Origin**		
Catalonia and rest of Spain	18	78
Rest of Europe	1	4
Latin America	4	18
**Highest educational level achieved**		
Secondary school	4	17
University degree	19	83
**Pregnancy risk**		
Low	5	22
Medium	5	22
High	9	39
Very high	4	17
**Gestational age in weeks (**$\overline{\boldsymbol{x}}=$**39.89 (***σ*= **1.27))**		
Preterm (< 38)	4	17
Term (> = 38)	19	83
**Type of birth**		
Vaginal	18	78
Instrumental	2	9
Caesarean section	3	13
**Onset of labour**		
Spontaneous	9	39
Induced	14	61
**Use of epidural anaesthesia**	22	96

After the analysis, three themes and six sub-themes emerged from the data (Table 3). The themes are set forth below with illustrative data using the participants’ own words. All quotations are suffixed by the participant number assigned to each woman, her age, the risk of her pregnancy, the kind of onset of labour and the type of birth.

**Table 3:** Themes and subthemes that emerged from the data

Theme	Sub-themes
Women’s low participation in shared decision-making	Women’s expectations for and obstacles to participation in shared decision-making
	Lack of clinician engagement with the birth plan
Lack of information provision for shared decision-making	Insufficient content
	Inappropriate timing
Suggestions to improve women’s participation in shared decision-making	Establishment of mutually respectful relationships between clinicians and women
	Continuity of coordinated, personalised perinatal care

### Women’s low participation in shared decision making

#### Women’s expectations for and obstacles to participation in shared decision making

Some women expressed that they did not make any decisions during their childbirth, and women who were willing to take an active role in decision-making encountered obstacles to achieving this. These women felt that they needed to be well prepared and active to be able to participate and described the experienced as a ‘fight’ to be involved in decision-making.

*“I feel that I didn’t decide anything. They [health professionals] decided.”* (W5, 42 years, medium-risk pregnancy, spontaneous onset, caesarean section)

*“I feel that if I hadn’t prepared myself so much, I would have had a much worse birth, because they wouldn’t have let me make decisions that I believe I decided because I fought.”* (W17, 30 years, high-risk pregnancy, spontaneous onset, vaginal birth)

*“I had the feeling of fighting from the beginning [ … ] with decisions that were being made where there was no other choice, but I couldn’t put more energy into imposing my will, you know, I wore myself out in that.”* (W2, 35 years, low-risk pregnancy, spontaneous onset, vaginal birth)

Other women expressed a high confidence in professionals and accepted a passive role in decision-making. Some of the women who took a passive role described themselves as “insufficiently prepared to make certain decisions”, as opposed to professionals, who were described as the appropriate individuals to make them. Some women with high-risk pregnancies felt that they had less space to participate in SDM and they were more steered to follow hospital protocols.

*“I came here [hospital] and I let myself go … ‘Do whatever you have to do, because you are professionals, I trust in you’”*. (W10, 31 years, low-risk pregnancy, spontaneous onset, vaginal birth)

*“Like when you go to an architect and the architect designs your home, you don’t question every step. [ … ] I said: ‘I trust my gynaecologist, for she has studied, and she knows’”.* (W7, 41 years, medium-risk pregnancy, onset by induction, vaginal birth)

*“I would rather have had a natural birth, but I didn’t have the chance to choose, I didn’t choose the caesarean, nor the induction … According to the protocol, I had to undergo induction [ … ] I thought, ‘With my age, with diabetes, I am not going to argue anything’ [ … ] If there is a possibility to choose, they should demonstrate it to you, because if not, you feel totally steered towards it.*” (W9, 40 years, high-risk pregnancy, onset by induction, caesarean)

#### Lack of clinician engagement with the birth plan

During focus groups, 16 women actively referred to their experience using the birth plan. They had completed birth plans with their partners and brought them to the hospital on the day of delivery. However, in almost all of the cases, health professionals did not ask them for their plan, and neither did they read it when the women or their partners offered it. Moreover, in high-risk pregnancies, their use was directly rejected by professionals.

“*I handed the birth plan from the week … I don’t know, very, very early. I was concerned, I studied it, I discussed it with my partner … It doesn’t matter, because it remained in the folder, just like it went into the hospital, it came out*.” (W2, 35 years, low-risk pregnancy, spontaneous onset, vaginal birth)

*“The birth plan is useless. Nobody read it, there were three shift changes, people who were there didn’t even know my name and by no means knew my birth plan.”* (W5, 42 years, medium-risk pregnancy, spontaneous onset, caesarean)

*“I underwent an induction, and it was like at the beginning, I couldn’t say anything [ … ] I had my birth plan, I wanted to do those things … nothing. They considered it was a birth with risk because of the weight of my baby.”* (W15, 30 years, very high-risk pregnancy, onset by induction, vaginal birth)

Sometimes, health professionals asked questions to women during childbirth instead of using the birth plan. This was described by some participants as adequate, as they thought that health professionals covered with their questions all the information needed for attending their childbirth. But other participants described that as insufficient due to professionals limiting these questions to asking if they wanted a “natural birth” or an “epidural”. Some participants justified this use of the birth plan, describing it as a document enabling women to be informed in advance about the different options offered by the hospital, rather than to be used during childbirth or enhancing their participation.

*“They asked me ‘Natural birth? OK!’ And they left the birth plan there. ‘Well, but within natural birth you can choose options.’ They didn’t look at it.”* (W17, 30 years, high-risk pregnancy, spontaneous onset, vaginal birth)

“*I understand that the birth plan is a tool so that at home you can think about it and have in mind what you want, and you can work on it with your partner.”* (W18, 35 years, high-risk pregnancy, spontaneous onset, vaginal birth)

### Lack of information provision for shared decision-making

#### Insufficient content

Most women described the information offered by health professionals at prenatal care, antenatal classes, and during birth, as insufficient to participate in the decision-making process. This lack of information was particularly notable regarding induction, in terms of the risks, duration, pain, and side effects; thus, women felt the need to search the internet or seek advice from other professionals or relatives.

*“I thought: ‘I feel so insecure about what is going to happen because I don’t have any information, so I have to search on Google.’ [ … ] I asked: ‘What is going to happen? Why … ? Which risks do I have … ? I asked every doctor I came across.*” (W8, 41 years, very high-risk pregnancy, onset by induction, vaginal birth)

In addition to individual appointments, many women obtained information from antenatal classes. Public community health centres and hospitals offer prenatal group classes led by midwives to every pregnant woman, regardless of pregnancy risk. Those classes were described as useful but sparse in content with regard to induction, breastfeeding, and postpartum; especially regarding inductions, women found a high disparity between expectations of birth created at these classes and their real experiences.

“*I feel induction is like another kind of birth and they only prepare you for a natural birth. So, I would have made more decisions, or I would have been more conscious of decisions to make, had I been better informed by professionals.*” (W19, 39 years, high-risk pregnancy, onset by induction, vaginal birth)

Some women felt neglected and uninformed when a complication arose during childbirth or there was a separation from the newborn. In some cases, professionals talked with each other without addressing the woman.

“*My childbirth [ … ] was complicated at the end but they didn’t explain to me why. I heard them saying that they were not going to give me the baby, but they didn’t explain anything to me. [ … ] A total lack of information.*” (W19, 39 years, high-risk pregnancy, onset by induction, vaginal birth)

#### Inappropriate timing

Participants reported that information had been given at inappropriate times, when they could not assimilate it to make informed decisions. This applied in the case of informed consent for several procedures, which participants reported not having had the opportunity to read and understand. Sometimes, information was given to women at the time of contractions, for instance concerning pain management, leaving them unable to decide or make a judgement on the information presented.

*“Sometimes they come up to you in the operating room and say, “Sign here”, but what validity does this have? It could have been signed by another woman.”* (W20, 32 years, very high-risk pregnancy, onset by induction, vaginal birth)

*“The midwife offered me a lot of different things for anaesthesia, eventually we decided epidural, but for a moment I thought, “Between contraction and contraction, making decisions about what kind of anaesthesia is the most appropriate … ” [...] You aren’t in the moment to decide.”* (W22*,* 37 years, very high-risk pregnancy, onset by induction, instrumental birth)

### Suggestions to improve women’s participation in shared decision-making

#### Establishment of mutually respectful relationships between clinicians and women

Professionals, partners and other family members were described as key people to promote women’s participation in decision-making during childbirth. Women only knew the professionals who attended their birth if they had happened to meet them at any of the prenatal appointments but knowing health professionals beforehand was highly valued by women to feel secure about the process.

*“‘Midwife 1’ attended me at one of the hospital appointments. And it was very important for me to see her in the delivery room. When she told me ‘I am ‘Midwife 1’, I thought ‘OK, everything is fine. Now I can give birth.’”* (W10, 31 years, low-risk pregnancy, spontaneous onset, vaginal birth)

Participants consider that professionals should improve the use of the birth plan, for example by extending the use of birth plans to high-risk pregnancies or creating a caesarean section plan, and increase their skills of good treatment, communication, and training to support women’s participation in shared decision-making.

*“What you need is a bit of empathy, they could ask you looking you in the face ‘Are you alright?’. They may hold your hand, calm you, say to you ‘Don’t worry, we are going to see what is happening’. They should work on non-verbal communication when they face a problem.”* (W22, 37 years, very high-risk pregnancy, onset by induction, instrumental birth)

“*During childbirth, they should follow step-by-step your emotional state. [ … ] They should use expressions like “How are you? How do you feel? What do you need? How can I help you? What do you want?”* (W2, 35 years, low-risk pregnancy, spontaneous onset, vaginal birth)

“*They should pay attention to your birth plan [ … ] and to make it more applicable, even when there is a high-risk pregnancy*.” (W20, 32 years, very high-risk pregnancy, onset by induction, vaginal birth) “*And a caesarean plan, it seems that women who undergo caesarean section have no choices, but they can also make decisions.*” (W17, 30 years, high-risk pregnancy, spontaneous onset, vaginal birth)

In regard to their partners, women expect them to be prepared and take part in the process of pregnancy and birth. HCB allows two people of women’s choice to support them during childbirth, which was highly appreciated by women. Moreover, women appreciate that partners or family members give them emotional support, remain calm and act, if it were to be necessary, as representatives of their will.

*‘I was feeling very lonely. But my mother came, and she helped me a lot, changed the sheets, helped me to vomit … I really liked that two people were allowed in the delivery room. For me, my mother was the light.’* (W2, 35 years, low-risk pregnancy, spontaneous onset, vaginal birth)

“*I almost lost consciousness but he [partner] was managing. Besides, he knew my preferences and she [midwife] had the knowledge about the options that there were [ … ]. They helped me at a time when I had so much pain that I wasn’t able to make decisions.*” (W6, 46 years, high-risk pregnancy, onset by induction, vaginal birth)

#### Continuity of coordinated, personalised perinatal care

Women felt that coordination among professionals within the hospital and also in community centres could be improved. For instance, transferring more information between shifts or in medical records. Also, participants proposed that “communication protocols” be created to standardise what information should be offered to women and when.

“*There isn’t a rule for explaining when to give information or not, I think it would be interesting to design protocols to say: “In this situation, we offer that information, in that situation, we give this other information” so that the patient’s experience doesn’t depend on what professional she meets.*” (W8, 41 years, very high-risk pregnancy, onset by induction, vaginal birth)

Women requested more tailored attention from professionals that could personalise and discuss hospital protocols on an individual basis. This could be achieved through the creation of mutual respect and a trusting relationship.

“*Why does a person who has known me for one day have to prescribe an induction on week 41? I needed the information to be only for me, with my context, with my characteristics and those of my baby [ … ] each birth is different, and we need individualised information.*” (W10, 31 years*,* low-risk pregnancy, spontaneous onset, vaginal birth)

“*If you don’t agree with hospital protocols [ … ] they [health professionals] should be more flexible. If there is a risk they should inform you, but without looking down on you, because maybe you are not making that decision knowing the risks that you are assuming.*” (W2, 35 years, low-risk pregnancy, spontaneous onset, vaginal birth)

In addition to coordination and personalisation, the women suggested that continuity of care both during childbirth and postpartum have scope for improvement. Some women highlighted that the lack of availability of midwives prevented them from feeling supported during childbirth. Moreover, shift changes were experienced as a sensitive moment. Besides, some women pointed out the need of having a visit during the postpartum period with some of the professionals that attended them, in order to solve doubts, share and understand information and have a complete narrative of their childbirth.

‘*They [health professionals] sent me to the room for the induction, and there, you are alone, you don’t have anyone. It was at night, and the nurses didn’t move from the counter until my husband went and said to them “Please, my wife is in labour, I can’t stand that”. And they came in and said, “Oh yes, she is in labour”. But, if he hadn’t, no one would have appeared.’* (W11, 35 years, high-risk pregnancy, onset by induction, vaginal birth)

*“When you know the professional and they make the shift change … I was afraid that she [Midwife] would look at me to see how everything was, that she would examine me, it made me feel scared. Even though you trust them a lot, you have this feeling.*” (W10, 31 years, low-risk pregnancy, spontaneous onset, vaginal birth)

“*I hoped that the midwife who attended my childbirth would come to explain to me what had happened [ … ] but nobody came to explain anything. One day, I came to the hospital to look for her and then she explained it to me. But if they had explained it to me when I was hospitalised, they would have saved me a lot of suffering, a lot of crying [ … ] and a lot of anxiety.*” (W5, 42 years, medium-risk pregnancy, spontaneous onset, caesarean)

## Discussion

This study explored women’s experiences of participation in SDM during hospital childbirth. Women had few experiences of and opportunities for participation in decision-making; thus, most decisions were made by others, considering neither the women’s needs and preferences for participation nor their birth plans. The information needed to take an active role during childbirth was perceived as missing or given to women at an inappropriate time, so their participation became less feasible. On the other hand, potential improvements that were identified as able to promote women’s participation were having a mutually respectful relationship with their care providers, the support of partners and other members of the family, and receiving continuity of a coordinated, personalised perinatal care.

This study showed that women who had expected to take an active role during childbirth, encountered barriers to doing so. Feeling out of control and little involved in medical decisions has been associated with a negative and traumatic experience of the birth process [38–40]. Some participants complied with medical decisions without the need to inquire about offered procedures, partly because they had a high level of trust in clinicians and described themselves as poorly prepared to make such decisions. To achieve an effective patient-provider partnership through SDM, it is needed for both professionals and patients to value the patient’s views, preferences, and expertise in their own lived process [41, 42]. In our study, some women who had been diagnosed with a high or very high-risk pregnancy seemed to have less confidence to question clinicians’ decisions and to participate in SDM, perhaps because they felt that if they made decisions other than those advised by health professionals they would be putting their babies and themselves at risk. Likewise, a recent metasynthesis found that women’s attitudes towards childbirth decisions are heavily affected by a medical diagnosis of “risk” [24].

The birth plan was experienced as a deficient method to promote women’s participation, largely because health professionals neither looked at it, nor took its content into account at the time of childbirth or conversations about decision-making. Instead, the birth plan was used to inform women about different birth care options that the hospital offered. This finding is consistent with other studies which have reported that the birth plan has been “institutionalised”, meaning that it is used as a hospital document to present service options rather than a document for women to express their preferences and needs [13–16]. Other uses of birth plans, complemented with other communication tools, should be explored in order to promote effective communication, thus enabling women’s participation in SDM [13].

Women reported that they often did not have sufficient information to make decisions in and about childbirth, despite their efforts to seek and gather this information during pregnancy and around the time of birth. Information and knowledge have been described as key factors for patient involvement in SDM [43]. Health professionals should draw on all of their knowledge and expertise in order to reduce knowledge asymmetry between women and clinicians and therefore facilitate women’s engagement in decision-making [44]. Specifically, the participants reported a lack of information provision with regard to birth complications and procedures during birth, such as induction, a finding which has been reported previously [45, 46]. Additionally, the women experienced a considerable shortage of information regarding informed consent before undergoing various procedures, such as an epidural or a caesarean section, and they highlighted the importance of information being given at an appropriate time. Some authors have highlighted that, even though obtaining informed consent during childbirth could be challenging, especially when an obstetric emergency arises [47], health professionals should persist in their efforts to inform women about the benefits and risks of obstetric practices in order to preserve their right to autonomy and self-determination [48–50]. Previous research has pointed out the importance of initiating an information exchange in antenatal care where there is sufficient time to explain and discuss the different options and to anticipate complex situations that may occur during birth [11]. For this purpose, various evaluated patient decision aids could be used during prenatal care [51, 52] to promote women’s involvement in SDM.

The interviewed women reported that health professionals should follow their emotional state and provide support when needed. The evidence shows that having a supportive environment facilitates patient participation in SDM [41] and women’s emotional well-being during childbirth could be considered as important for women as their physical health or that of their newborns [53, 54]. Effective tools should take into account these needs and promote means for women to convey their emotions and, ultimately, to increase the quality of care. Moreover, to promote women’s involvement in SDM, women suggested that professionals should improve their communication and relational skills. Relational and risk communication skills can help health professionals to effectively promote patient participation [55] and meet the emotional and communicative demands of childbearing women.

Finally, women stressed the need to receive continuous support and a coordinated, personalised care during pregnancy and childbirth. Evidence supports midwife-led continuity of care models as the best standard of care for pregnancy and childbirth [56–58]. Furthermore, coordination, continuity of care, and interdisciplinary teamwork has been highlighted as essential for a patient-centred care, a model that strongly promotes patients’ participation in SDM [59]. The study participants reported the low availability of midwives during hospital admission. A recent study from midwives’ perspectives suggested that Catalonian hospitals do not have sufficient resources to make midwife-led continuity of care feasible, and also that some hospitals present a highly hierarchical work environment that hampers coordination [60].

Fulfilment of women’s expectations of participation should be a priority of maternity health services. A comprehensive approach to facilitating women’s involvement in SDM during hospital childbirth should include a training programme for health professionals and women, accompanied by an effective communication tool to enhance women’s participation. An example of an effective training programme for women was an educational intervention implemented during prenatal classes. It aimed to reduce rates of elective induction of labour by providing information to women and empowering them to initiate conversations about risks and alternatives with health professionals [61, 62]. Our study demonstrates that the use of only a single tool, such as the birth plan, as least in its current format and implementation, is insufficient to promote women’s participation as long as the knowledge asymmetry remains, and stakeholders are poorly prepared.

### Limitations and further research

To our knowledge, this is the first study that explores women’s experiences of participation in SDM during hospital childbirth in Spain. The qualitative methodology provided a deep and broad insight into women’s experiences, however, this study has intrinsic limitations. Although focus groups allowed for rich interactions among participants and fostered discourse around participation in SDM during childbirth, an uncommon topic to most women in our context, the data gathering using interviews in addition to focus groups could have provided deeper understanding about women’s experiences, but this was beyond the possibilities of this study. As data was collected from women who attended one particular hospital in Spain, their experiences may not represent the vast majority of women giving birth in the country. Moreover, the sample was self-selecting and, therefore, women with a higher educational level were over-represented. Thus, extrapolation of findings should be done cautiously. Besides, due to convenience sampling, there is a risk that women with less satisfaction regarding their involvement in SDM were overly represented in the study. However, their experiences may provide relevant lessons as opportunities for improvement concerning this topic.

Although this research identified some specific and crucial decisions in which women wanted to be involved, such us undergoing an induction or the methods of pain management to use, more research is needed to define which decisions are the most important for women to participate in, also taking into account professionals’ perspectives. Moreover, further research should include women with lower socio-economic status, different origins, and mental or physical disabilities and take into account partners’ views and their experiences of involvement in SDM during childbirth. Furthermore, individual interviews with women would provide deeper insights on women’s narratives and would overcome possible effects of peer pressure. These findings should help to rethink birth plans and increase their real-world value. Besides, this information would facilitate the development, implementation, and evaluation of appropriately contextualised interventions to truly promote women’s participation in SDM during hospital childbirth.

### Implications for policy and practice

Clinical implications of this work include the need to develop strategies to promote women’s participation in SDM, such as improving the use of birth plans and the further development of other communication tools. When given the opportunity to complete a birth plan, women and their families may form expectations about participation and involvement. However, according to the participants, the use of birth plans thus far amounts to mere procedure, with no clinical significance. Health professionals could explore birth plans as a tool to further involve women, and help them to revisit decisions if the childbirth takes a different course. Furthermore, birth plans could be complemented with the use of other communication tools, such as patient decision aids [63]. Additionally, in the context of this study, birth plans were not amended for women with high-risk pregnancies. Promoting means of participation for women diagnosed with high-risk pregnancies should be a priority, considering the demographic characteristics of the pregnant women population in Spain [64]. Policy-makers should foster the implementation and evaluation of interventions to promote women’s participation in SDM during childbirth, which would include training programmes and changes in organisational models to trigger an ideological shift from paternalistic healthcare to an increasingly participation-based healthcare.

## Conclusions

This study has shown that women who were willing to take an active role in SDM during hospital childbirth faced difficulties in doing so. The information needed to take an active role during childbirth was perceived as missing or given to women at an inappropriate time. Potential improvements identified as enablers of women’s participation were having a mutually respectful relationship with their care providers, the support of partners and other members of the family, and receiving continuity of a coordinated and personalised perinatal care. Enhancing women’s participation requires the acquisition of skills by health professionals and women and the development, implementation, and evaluation of interventions to facilitate women’s engagement in SDM.

## Data Availability

The datasets used and/or analysed during the current study are available from the corresponding author on reasonable request.

## Abbreviations

SDM: Shared decision-making
HCB: Hospital Clinic of Barcelona
